# Impact of malnutrition on health-related quality of life in persons receiving dialysis: a prospective study

**DOI:** 10.1017/S000711452100249X

**Published:** 2022-06-14

**Authors:** Daniela Viramontes-Hörner, Zoe Pittman, Nicholas M. Selby, Maarten W. Taal

**Affiliations:** 1 Centre for Kidney Research and Innovation, Academic Unit for Translational Medical Sciences, School of Medicine, University of Nottingham, Royal Derby Hospital, Uttoxeter Rd, Derby DE22 3NE, UK; 2 Department of Renal Medicine, University Hospitals of Derby and Burton NHS Foundation Trust, Royal Derby Hospital, Uttoxeter Rd, Derby DE22 3NE, UK

**Keywords:** Dialysis, Malnutrition, Quality of life, Subjective Global Assessment

## Abstract

Health-related quality of life (HRQoL) is severely impaired in persons receiving dialysis. Malnutrition has been associated with some measures of poor HRQoL in cross-sectional analyses in dialysis populations, but no studies have assessed the impact of malnutrition and dietary intake on change in multiple measures of HRQoL over time. We investigated the most important determinants of poor HRQoL and the predictors of change in HRQoL over time using several measures of HRQoL. We enrolled 119 haemodialysis and thirty-one peritoneal dialysis patients in this prospective study. Nutritional assessments (Subjective Global Assessment (SGA), anthropometry and 24-h dietary recalls) and HRQoL questionnaires (Short Form-36 (SF-36) mental (MCS) and physical component scores (PCS) and European QoL-5 Dimensions (EQ5D) health state (HSS) and visual analogue scores (VAS)) were performed at baseline, 6 and 12 months. Mean age was 64 (14) years. Malnutrition was present in 37 % of the population. At baseline, malnutrition assessed by SGA was the only factor independently (and negatively) associated with all four measures of HRQoL. No single factor was independently associated with decrease in all measures of HRQoL over 1 year. However, prevalence/development of malnutrition over 1 year was an independent predictor of 1-year decrease in EQ5D HSS, and 1-year decrease in fat intake independently predicted the 1-year decline in SF-36 MCS and PCS, and EQ5D VAS. These findings strengthen the importance of monitoring for malnutrition and providing nutritional advice to all persons on dialysis. Future studies are needed to evaluate the impact of nutritional interventions on HRQoL and other long-term outcomes.

Health-related quality of life (HRQoL) is one of the most important and widely used patient-centred outcome measures in renal research and clinical settings that provides information about an individual’s well-being with respect to physical, mental, social and somatic domains of health^(1)^. HRQoL is severely impaired in persons receiving dialysis compared with the general population^(2)^, and decreased HRQoL has been associated with increased number of hospitalisations and poor survival in persons receiving haemodialysis (HD) and performing peritoneal dialysis (PD)^(3,4)^. Several factors have been identified as important determinants of poor HRQoL in persons on dialysis, including older age, female sex, unemployment, lack of educational qualifications, anaemia, presence of diabetes and other co-morbidities, lack of sleep, depression and poor nutritional status^(2,5–7)^.

Malnutrition is a common and major complication, as well as an independent risk factor for increased mortality in the dialysis population^(8)^. Several terms for referring to malnutrition have been used in both the renal dietetic practice and research. However, in 2008, the International Society of Renal Nutrition and Metabolism suggested a single term, ‘Protein-energy wasting’^(9)^, which has improved communication and clarified thinking across renal multidisciplinary care teams. For the purpose of this study, the term ‘malnutrition’ will be used as a synonymous with ‘Protein-energy wasting’. The pathogenesis of malnutrition is complex and results from the interaction of several factors such as loss of appetite causing poor nutritional intake, loss of protein and micronutrients during dialysis, increased inflammation and oxidative stress, presence of co-morbidities and decreased physical activity^(10)^. Previous cross-sectional analyses have reported that HRQoL, as assessed by the thirty-six-Item Short Form Health Survey (SF-36), Kidney Disease Quality of Life Short Form or the European Quality of Life 5-Dimensions (EQ5D) questionnaire, was significantly lower in malnourished persons on dialysis compared with those who were well-nourished^(5,6,11–15)^. However, none of these studies included a comprehensive assessment of dietary intake, and all used a single instrument to assess HRQoL. Hence, further evidence is needed regarding the impact of malnutrition and dietary intake on HRQoL.

It has been previously reported that HRQoL declines over time in persons receiving dialysis^(16,17)^, but factors that contribute to changes in HRQoL over time, in particular measures of nutritional status, have not been adequately investigated. We therefore sought to determine the most important determinants of poor HRQoL, as well as the predictors of change in HRQoL over time in persons receiving dialysis in a prospective study, with a particular focus on dietary intake and malnutrition.

## Materials and methods

### Patient population

One hundred nineteen HD and thirty-one PD patients who were ≥18 years of age, had a dialysis vintage >3 months or were starting either HD or PD treatment, and were able to give written informed consent were enrolled in this 1-year single-centre prospective observational study conducted in the Department of Renal Medicine, Royal Derby Hospital. Recruitment was from September 2016 to August 2017. Persons receiving HD used high-flux polysulphone, polyarylethersulfone or polyvinylpyrrolidone dialyzers and were dialyzed at least three times per week for 3–4 h. Persons performing PD used lactate/bicarbonate-buffered 1·36 and 3·86 % glucose (Physioneal; Baxter®), 7·5 % icodextrin (Extraneal; Baxter®) and/or 1·1 % amino acid-containing solutions (Nutrineal; Baxter®). The exclusion criteria were pregnancy or intending pregnancy, breast-feeding and hospitalisation at the time of recruitment. This study was conducted according to the guidelines laid down in the Declaration of Helsinki, and all procedures involving patients were approved by the local Research Ethics Committee (East Midlands – Nottingham 1. REC reference: 16/EM/0243). Written informed consent was obtained from all patients.

### Sociodemographic and medical characteristics

Baseline sociodemographic characteristics including chronological age, sex, ethnicity, educational level and employment status, as well as present co-morbidities, history of CVD, blood results and time since first dialysis treatment (i.e. dialysis vintage) were collected from direct interview and/or electronic medical records.

### Nutritional assessments

At baseline, 6 and 12 months, we conducted the following detailed nutritional assessments:

#### Dietary intake

Twenty-four-hour dietary recalls were used for dietary intake assessment. From each participant, an experienced dietitian collected precise and comprehensive information regarding food and drink intake during a 24-h period. In persons receiving HD, 24-h dietary recalls included information from a dialysis day, a non-dialysis day and a weekend day, while in persons performing PD, dietary recalls obtained information from two weekdays and one weekend day. We used the software Dietplan 7 (Forestfield Software Limited) to calculate the average intake of energy content, protein and fat. Average energy and protein intake were then expressed in daily kcal and g, respectively, per kg of ideal body weight.

#### Anthropometry

International standards for anthropometric assessment^(18)^ were followed to measure post-dialysis weight, height, mid-arm circumference and triceps skinfold thickness. Weight and height were used to calculate BMI (reported in kg/m^2^). Mid-arm muscle circumference was calculated using the following equation: mid-arm muscle circumference (cm^2^) = mid-arm circumference – (3.14 × triceps skinfold thickness), where mid-arm circumference and triceps skinfold thickness were measured in cm.

#### Handgrip strength

We used the Takei 5401 handgrip digital dynamometer (Takei Scientific Instruments Co. Ltd) to measure handgrip strength (HGS) within the first hour of HD treatment or during PD clinic visits. HGS measurement was conducted in the non-fistula arm or the dominant arm if this did not have a fistula as previously described^(19)^.

#### Subjective Global Assessment

An experienced dietitian conducted the validated seven-point scale Subjective Global Assessment (SGA)^(20,21)^ for the assessment of nutritional status. The seven-point scale SGA is composed of six elements (weight change, dietary intake, gastrointestinal symptoms, functional capacity, co-morbidities and physical examination), which are scored between 1 and 7 in order to determine the overall SGA score. The lower the overall SGA score, the more severe the degree of malnutrition. For baseline analysis, participants were classified as being well-nourished (SGA scores 6–7) or malnourished (SGA score ≤ 5). For further analysis, participants who completed 12 months of follow-up were classified according to their nutritional status over 1 year into two groups: (a) ‘stayed or became well-nourished’ – participants who were well-nourished throughout the 1 year or became well-nourished at either 6 or 12 months (i.e. malnourished at baseline but well-nourished at 6 or 12 months); (b) ‘stayed or became malnourished’ – participants who were malnourished throughout the 1 year or who became malnourished at either 6 or 12 months (i.e. well-nourished at baseline but malnourished at 6 or 12 months). As part of their routine clinical care, all malnourished patients received dietetic advice by their usual renal dietitian, which may have included the use of nutritional supplements; however, we did not assess the impact of specific nutritional supplements in our analyses.

### Quality of life assessments

HRQoL was assessed at baseline, 6 and 12 months using the SF-36 survey and the EQ5D questionnaire, which are validated and standardised instruments that have been widely used to assess HRQoL in the general and dialysis populations^(11,22–24)^.

The SF-36 survey comprises thirty-six questions that assess eight health state domains: physical functioning, role physical, bodily pain, general health, vitality, social functioning, role emotional and mental health. These eight domains are then summarised into two scores: the physical component score (PCS) and the mental component score (MCS)^(22)^. Both the PCS and MCS were calculated according to well-defined guidelines^(25–27)^. In brief, ten questions of the SF-36 survey were first recoded so that a higher score represented a better health state (e.g. question no. 7 regarding bodily pain was recoded so that a high score indicated no pain at all). Next, raw scores for each health state domain were calculated by summing across items in the same health state domain (e.g. role physical = scores from questions 4a + 4b + 4c + 4d), and then raw scores were transformed to a 0–100 scale^(25)^. Each of the eight SF-36 transformed scales was then standardised using a *z*-score transformation and the means and standard deviations from the general UK population^(26)^. Then, the PCS and MCS were calculated by multiplying each scale *z*-score by their respective physical and mental factor score coefficients and summing the eight products. Finally, both the PCS and MCS were standardised to a T-score by multiplying by 10 and adding the resultant product to 50^(27)^. A PCS or MCS score above or below 50 is therefore above or below the average for the general population.

The EQ5D questionnaire consists of a health state score (HSS) and a visual analogue score (VAS). The HSS comprises five dimensions (i.e. mobility, self-care, usual activities, pain/discomfort and anxiety/depression) with five available response levels (i.e. no, slight, moderate, severe and extreme problems/unable to). The HSS is calculated using specific coefficients for the five dimensions and response levels as described elsewhere^(23)^, and it ranges from −0·285 (for the worst health state) to 1 (for the best health state). The VAS uses a thermometer-like scale numbered from 0 to 100 to grade the current health status of individuals; the higher the VAS the better the health state.

### Statistical analyses

All statistical analyses were conducted using the statistical software SPSS version 25.0 (IBM Corporation). Continuous variables are presented as mean (standard deviation) or median (interquartile range), while categorical variables are presented as percentages. Missing data were omitted (C reactive protein, *n* 7 and HGS, *n* 6). Paired *t* test and Wilcoxon test were used for intragroup comparisons in the case of continuous variables. Student’s *t* test and Mann–Whitney *U* test were used for intergroup comparisons for continuous variables and *χ* ^2^ test or Fisher’s exact test for categorical variables. To determine the significance and strength of associations between continuous variables, we used Pearson’s and Spearman’s correlation coefficients. Multivariable linear regression analyses were performed to identify the independent determinants associated with HRQoL at baseline. Adjusted *R* ^2^, unstandardised (B) and standardised (Beta) coefficients were reported.

Change in HRQoL over 1 year was defined as a five-point change (increase or decrease) in the SF-36 MCS, SF-36 PCS and EQ5D VAS, and a 0·037 change (improvement or deterioration) in the EQ5D HSS. These thresholds represent the Minimally Important Difference defined as the smallest change in the HRQoL score of interest which a patient perceives as meaningful or beneficial^(28)^. In terms of supporting the interpretability of the change in HRQoL, it has been suggested that using the Minimally Important Difference is better than using the clinically important difference (i.e. change or difference associated with outcomes), though these are in fact similar^(28–31)^. For statistical analysis, participants were grouped into those with an increase in or stable HRQoL scores over time *v*. a decrease in HRQoL scores. Multivariable logistic regression analyses were conducted to identify the independent predictors of increased/stable HRQoL *v*. decreased HRQoL over 1 year. Nagelkerke *R* ^2^ for the models and Hosmer and Lemeshow test *P*-value were reported.

Independent variables included in the multivariable linear and logistic regression analyses were selected on the basis of significant associations in univariable analyses or biological plausibility (i.e. chronological age, sex and employment status). For all statistical analyses, a *P*-value <0·05 was considered to have statistical significance.

Our original sample size determination was performed for an observational study with mortality as the primary outcome^(32)^. However, for the purpose of this analysis, we conducted a retrospective sample size calculation with decrease in MCS, PCS, HSS and VAS as the primary outcomes. With a sample size of 117 participants split into two groups (Group 1: stayed well-nourished + became well-nourished over 1 year, *n* 90; Group 2: stayed malnourished + became malnourished over 1 year, *n* 27), the analysis would hypothetically have had 80 % power to detect OR of 3·45, 3·47, 3·57 and 3·51 for the decrease in MCS, PCS, HSS and VAS, respectively (STATA, version 16.1; StataCorp LLC).

## Results

### Baseline participant characteristics

Baseline characteristics of 119 HD and thirty-one PD participants are summarised in Table 1. Mean age of the whole study population was 64 (14) years. Thirty-six percent of the participants were female, and 41 % had been diagnosed with diabetes. The majority of the participants were White British (88 %), unemployed or retired (75 %) and had some level of education (57 %). Malnutrition (as determined by seven-point SGA) was present in 37 % of the population. Mean PCS and MCS were 25·4 (13·1) and 47·4 (12·1), respectively, which were lower than values for healthy UK volunteers aged 18–64 years (i.e. 50 (10) for both scores)^(27)^. EQ5D HSS (0·742, interquartile range 0·494–0·873) and VAS (60, interquartile range 49·8–80) were also lower than that reported for the general UK population (*n* 3381; HSS 0·86 (0·23), VAS 82·5 (16·9))^(33,34)^.

**Table 1.** Baseline participant characteristics including demographics, clinical, biochemical, nutritional and health-related quality of life scores (Numbers and percentages; median values and interquartile range (IQR); mean values and standard deviations)

Variable	*n* 150	
	*n* / mean	% / sd
Age (years)	64	14
Female	54	36
White British	132	88
Educational qualifications	85	57
Unemployed/retired	113	75
Diabetes	61	41
CHD	60	40
Malnutrition	55	37
Dialysis vintage (months)		
Median	29	
IQR	10–68	
Hb (g/l)	117	13
Serum albumin (g/l)	31·6	4·5
C reactive protein (mg/l)		
Median	8	
IQR	4–17	
Total cholesterol (mmol/l)	4·1	1·2
Serum creatinine (µmol/l)	647	214
Serum phosphate (mmol/l)	1·56	0·51
Serum potassium (mmol/l)	4·6	0·7
Energy intake (kcal/kg per d)	21·0	7·6
Protein intake (g/kg per d)	0·88	0·29
Fat intake (g/d)	58·1	29·8
Post-dialysis weight (kg)	79·4	20·8
BMI (kg/m^2^)	27·7	6·3
Handgrip strength (kg)	23·1	11·5
Mid-arm muscle circumference (cm^2^)	25·6	3·8
Triceps skinfold thickness (mm)	17·2	7·2
SF-36 Mental component score	47·4	12·1
SF-36 Physical component score	25·4	13·1
EQ5D Health state score		
Median	0·742	
IQR	0·494–0·873	
EQ5D Visual analogue score		
Median	60	
IQR	49·8–80	

EQ5D, European Quality of Life 5-Dimensions; SF-36, 36-Item Short Form Health Survey.

### Determinants of health-related quality of life

Table 2 shows associations with HRQoL at baseline in univariable analysis. Only malnutrition assessed by SGA was strongly associated with worse scores in all HRQoL measures in comparison with those participants who were well-nourished. Additionally, SGA score showed strong positive correlations with all HRQoL scores. Unemployed/retired participants and those with diabetes had lower PCS and both EQ5D scores compared with employed participants and those without diabetes, respectively. CHD, longer dialysis vintage and being on HD were associated with lower PCS and EQ5D HSS. Age was positively associated with MCS and EQ5D HSS. Males showed higher PCS in comparison with females. Lower C reactive protein and higher serum albumin and serum creatinine were associated with higher PCS. Other markers of nutritional status including protein intake and HGS were associated with two of the four HRQoL measures.

**Table 2.** Determinants of health-related quality of life in univariable analysis at baseline in persons receiving dialysis (Mean values and standard deviations; median and interquartile range (IQR))

Factor	Dialysis patients (*n* 150)											
	Mental component score			Physical component score			EQ5D Health state score			EQ5D visual analogue score		
	Mean	sd	*P*	Mean	sd	*P*	Median	IQR	*P*	Median	IQR	*P*
Sex			0·1			0·005			0·07			0·7
Female (*n* 54)	45·2	12·0		21·5	12·0		0·697	0·419–0·816		60	50–75	
Male (*n* 96)	48·6	12·1		27·6	13·3		0·781	0·590–0·879		62·5	45–80	
Malnutrition defined by SGA			<0·0001			<0·0001			<0·0001			<0·0001
Yes (*n* 55)	41·8	11·1		17·7	9·7		0·524	0·305–0·727		50	30–60	
No (*n 9*5)	50·6	11·6		29·9	12·9		0·816	0·662–0·892		70	50–80	
Diabetes			0·3			0·002			<0·0001			0·04
Yes (*n* 61)	46·2	12·8		21·4	11·9		0·650	0·352–0·795		50	32·5–75	
No (*n* 89)	48·2	11·7		28·2	13·3		0·801	0·644–0·879		65	50–80	
CHD			0·9			0·02						0·3
Yes (*n* 60)	47·5	12·2		22·3	12·6		0·700	0·390–0·808	0·03	60	41·3–75	
No (*n* 90)	47·3	12·2		27·5	13·2		0·789	0·595–0·879		62·5	50–80	
Dialysis modality			1			0·007						0·2
Haemodialysis (*n* 119)	47·3	12·3		24·0	13·0		0·704	0·454–0·861	0·01	60	45–75	
Peritoneal dialysis (*n* 31)	47·4	11·7		31·0	12·4		0·803	0·699–0·879		65	50–80	
Educational qualifications			0·6			0·3			0·7			0·2
Yes (*n* 85)	47·8	12·7		26·4	12·9		0·777	0·520–0·872		65	50–80	
No (*n* 65)	46·8	11·4		24·2	13·4		0·727	0·475–874		55	45–77·5	
Employed												
Yes (*n* 37)	47·9	11·2	0·8	31·8	14·4	0·001	0·801	0·687–0·907	0·04	80	50–85	0·004
No (*n* 113)	47·2	12·5		23·3	12·1		0·725	0·468–0·864		60	45–75	

	Pearson’s *r*		*P*	Pearson’s *r*		*P*	Spearman’s Rho		*P*	Spearman’s Rho		*P*
Age (years)	0·371		<0·0001	0·058		0·5	0·214		0·009	0·146		0·08
SGA score	0·332		<0·0001	0·473		<0·0001	0·484		<0·0001	0·392		<0·0001
Dialysis vintage (months)	0·056		0·5	–0·183		0·03	–0·165		0·04	0·05		0·5
C reactive protein (mg/l)	0·094		0·3	–0·187		0·03	–0·137		0·1	–0·102		0·2
Hb (g/l)	0·04		0·6	0·122		0·1	0·069		0·4	0·081		0·3
Serum creatinine (µmol/l)	–0·075		0·4	0·193		0·02	0·114		0·2	0·141		0·09
Serum albumin (g/l)	0·091		0·3	0·171		0·04	0·142		0·08	0·128		0·1
Total cholesterol (mmol/l)	–0·11		0·2	0·06		0·5	0·105		0·2	0·098		0·2
Energy intake (kcal/d)	0·085		0·3	0·115		0·2	0·162		0·05	0·084		0·3
Protein intake (g/d)	0·102		0·2	0·167		0·04	0·175		0·03	0·117		0·2
Fat intake (g/d)	0·042		0·6	0·045		0·6	0·071		0·4	–0·014		0·9
BMI (kg/m^2^)	0·029		0·7	–0·002		1	–0·007		0·9	–0·009		0·9
Handgrip strength (kg)	–0·014		0·9	0·361		<0·0001	0·279		<0·0001	0·107		0·2
MAMC (cm^2^)	–0·013		0·9	0·051		0·5	0·002		1	0·033		0·7

EQ5D, European Quality of Life 5-Dimensions; MAMC, mid-arm muscle circumference; SGA, Subjective Global Assessment.

In multivariable linear regression analyses (Table 3), nutritional status was the only determinant independently associated with all four HRQoL measures at baseline, such that malnutrition was associated with lower scores. Diabetes was an independent determinant of decreased PCS and both EQ5D scores, whereas being unemployed or retired was independently associated with lower PCS and EQ5D VAS. Older age was found to be an independent determinant of better MCS and EQ5D HSS, while being on HD showed an independent association with worse PCS and EQ5D HSS. In another multivariable model that included SGA as a continuous variable, a low SGA score was independently associated with worse HRQoL in all four measures (online Supplementary Table 1).

**Table 3.** Multivariable linear regression analysis to identify independent determinants of health-related quality of life at baseline (Unstandardised (B) and standardised (Beta) coefficients)

Independent variables	Dependent variable											
	Mental component score			Physical component score			EQ5D Health state score			EQ5D Visual analogue score		
	B	Beta	*P*	B	Beta	*P*	B	Beta	*P*	B	Beta	*P*
Age (years)	0·334	0·373	<0·0001	0·092	0·094	0·2	0·005	0·259	0·002	0·230	0·138	0·1
Sex (Female *v*. Male)	–0·626	–0·025	0·8	–0·633	–0·023	0·8	–0·075	–0·126	0·2	–5·026	–0·106	0·3
Unemployed/retired (Yes *v*. No)	–2·278	–0·082	0·3	–4·603	–0·153	0·04	–0·051	–0·079	0·3	–9·314	–0·181	0·03
Dialysis modality (HD *v*. PD)	–0·624	–0·021	0·8	–4·937	–0·154	0·03	–0·117	–0·171	0·02	–6·113	–0·112	0·2
Nutritional status (Malnourished *v*. Well-nourished)	–7·984	–0·316	<0·0001	–10·68	–0·389	<0·0001	–0·225	–0·384	<0·0001	–19·32	–0·410	<0·0001
Diabetes (Yes *v*. No)	–1·202	–0·049	0·5	–6·166	–0·231	0·002	–0·151	–0·264	<0·0001	–9·047	–0·198	0·01
Dialysis vintage (months)	0·013	0·070	0·4	–0·017	–0·086	0·2	0·000	–0·064	0·4	0·019	0·055	0·5
Handgrip strength (kg)	–0·009	–0·009	0·9	0·173	0·150	0·1	0·005	0·184	0·06	–0·093	–0·047	0·7
Adjusted *R* ^2^	0·212			0·341			0·352			0·223		

EQ5D, European Quality of Life 5-Dimensions; HD, haemodialysis; PD, peritoneal dialysis.

### Predictors of change in health-related quality of life

**Fig. 1.**
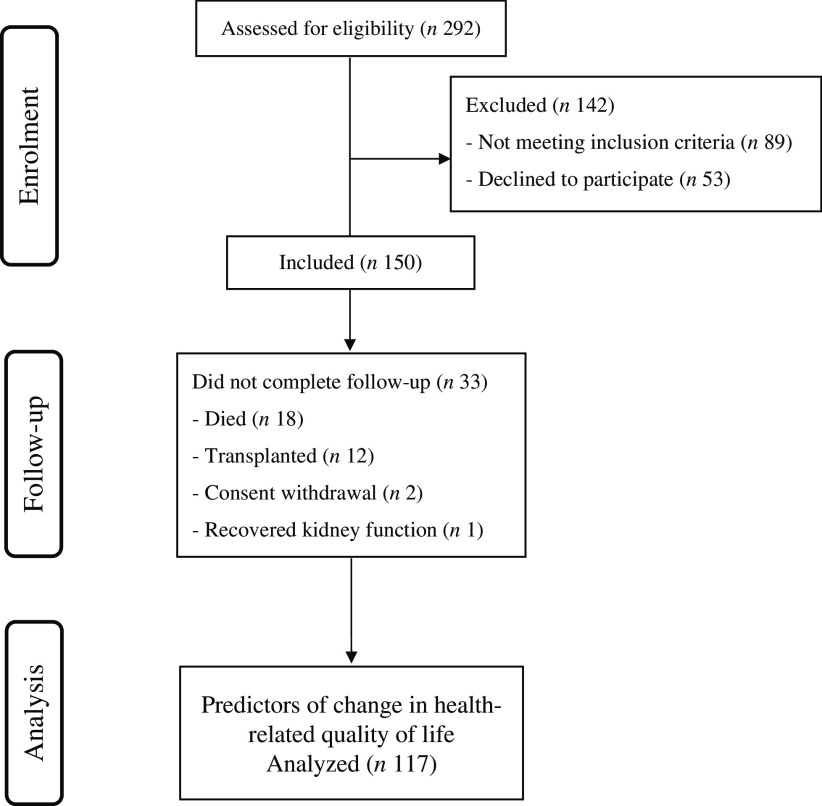
The Consolidated Standards of Reporting Trials (CONSORT) flow chart of participant progression through the study.

During follow-up, eighteen participants died, twelve received a kidney transplant, two withdrew their consent and one recovered kidney function sufficiently to discontinue dialysis. Thus, 117 participants completed 1 year of follow-up (Fig. 1). There were no significant changes in mean MCS and PCS or median EQ5D VAS at 12 months compared with baseline (47·6 (12·1) *v*. 46·5 (12·9), 25·7 (12·5) *v*. 24·1 (13·5), 60 (50 to 77·5) *v*. 55 (40 to 75); *P* > 0·05 for all comparisons); however, median EQ5D HSS decreased significantly at 1 year in comparison with baseline (0·751 (0·539 to 0·879) *v*. 0·718 (0·390 to 0·877); *P* = 0·02).

Univariable analysis showed that participants who stayed or became malnourished during 1 year (*n* 27) were more likely to evidence a decrease in EQ5D HSS (70 % *v*. 43 %; *P* = 0·01) at 12 months compared with those who stayed or became well-nourished during 1 year (*n* 90). Univariable analysis also showed that those participants who had a decrease in energy and fat intake over 1 year had a decrease in three of the four HRQoL measures at 12 months compared with those who had an increase in energy and fat intake over 1 year. Additionally, 1-year decrease in serum total protein and Hb was associated with the 1-year decline in PCS. Furthermore, participants with CHD evidenced a greater proportion with the 1-year decrease in MCS and EQ5D VAS, while lack of educational qualifications was associated with the 1-year decline in EQ5D VAS. No associations were observed with other potential risk factors, including chronological age, sex, employment status, presence of diabetes and dialysis modality (online Supplementary Table 2).

Table 4 summarises the multivariable logistic regression analyses to identify independent predictors of decrease in HRQoL over 1 year. No single factor was independently associated with decrease in all measures of HRQoL. However, prevalence or development of malnutrition over 1 year was an independent predictor of the 1-year decrease in EQ5D HSS and a decrease in fat intake over 1 year independently predicted the 1-year decline in MCS, PCS and EQ5D VAS. Lack of educational qualifications and presence of CHD each independently predicted a decrease in EQ5D VAS.

**Table 4.** Multivariable logistic regression analyses showing independent predictors of decrease in health-related quality of life scores over 1 year *v*. increase/stable health-related quality of life scores (Odds ratio and 95 % confidence intervals)

Predictor	Dependent variable											
	Decrease in MCS			Decrease in PCS			Decrease in EQ5D HSS			Decrease in EQ5D VAS		
	OR	95 % CI	*P*	OR	95 % CI	*P*	OR	95 % CI	*P*	OR	95 % CI	*P*
Sex (Female *v*. Male)	1·45	0·61, 3·48	0·4	0·82	0·36, 1·87	0·6	1·47	0·65, 3·34	0·4	1·06	0·46, 2·45	0·9
Educational qualifications (No *v*. Yes)	0·81	0·35, 1·86	0·6	1·11	0·49, 2·48	0·8	1·87	0·85, 4·14	0·1	2·40	1·06, 5·41	0·04
CHD (Yes *v*. No)	2·16	0·94, 4·97	0·07	0·98	0·43, 2·25	1·0	1·59	0·70, 3·58	0·3	2·37	1·03, 5·47	0·04
Nutritional status over 1 year (Stayed or became malnourished *v*. stayed or became well-nourished)	1·87	0·73, 4·81	0·2	0·84	0·32, 2·16	0·7	3·04	1·16, 7·98	0·02	1·99	0·76, 5·20	0·2
1-year decrease in serum total protein (Yes *v*. No)	1·98	0·88, 4·46	0·1	2·16	0·98, 4·79	0·06	1·27	0·58, 2·75	0·6	0·84	0·37, 1·89	0·7
1-year decrease in fat intake (Yes *v*. No)	2·72	1·20, 6·18	0·02	2·29	1·03, 5·08	0·04	1·81	0·82, 3·98	0·1	2·77	1·23, 6·22	0·01
Nagelkerke R^2^	0·167			0·107			0·154			0·209		
Hosmer and Lemeshow test *P* Value	0·168			0·595			0·924			0·765		

EQ5D, European Quality of Life 5-Dimensions; HSS, Health State Score; MCS, Mental Component Score; PCS, Physical Component Score; VAS, Visual Analogue Score.

## Discussion

In this prospective study, we observed that the presence of malnutrition was the most consistent independent determinant of decreased HRQoL as assessed by both the SF-36 and EQ5D in persons on dialysis at baseline. Additionally, prevalence/development of malnutrition over 1 year was an independent predictor of the 1-year decrease in EQ5D HSS and a decrease in fat intake (a marker of deteriorating nutritional intake) independently predicted decreases in MCS, PCS and EQ5D VAS.

Malnutrition is one of the major and most frequent complications observed in persons receiving dialysis that is also often underrecognised and neglected. It is clinically important because it is associated with poor survival and decreased HRQoL^(8)^. The relationship between malnutrition and decreased HRQoL in the dialysis population has been previously investigated only in cross-sectional analyses. Gunalay *et al.* ^(11)^ observed that malnourished persons on HD and performing PD had significantly lower EQ5D scores (both HSS and VAS) compared with those who were well-nourished. A cross-sectional analysis of the Convective Transport Study reported that a higher SGA score was independently associated with higher SF-36 PCS and MCS, after adjusting for covariates^(13)^. Several other studies have also reported that a low SGA score and/or a high Malnutrition Inflammation Score (a modified version of the SGA) correlate with lower SF-36 PCS and MCS^(5,6,12,14)^. Our study adds to published data by showing that malnutrition at baseline was an independent determinant of decreased HRQoL across all domains and using two different measures (SF-36 and EQ5D), whereas previous studies have used only one measure or have reported associations with some but not all measures^(15)^. Moreover, our analysis was adjusted for other important determinants of HRQoL including chronological age, sex, presence of diabetes, employment status, dialysis modality, dialysis vintage and HGS. Additionally, we have confirmed an association between the severity of malnutrition and HRQoL as shown by the strong and independent positive correlation between SGA score and all HRQoL scores.

We observed that EQ5D HSS (which includes physical and psychosocial variables) decreased over 1 year in the whole cohort, though no change in mean MCS and PCS or median EQ5D VAS was observed. This may be in part because participants with decreasing HRQoL may have been more likely to die during the observation period. Previous prospective studies have reported that persons on dialysis experience a decline in the physical and mental components of HRQoL over time^(16,17)^; however, they did not explore the factors associated with this decrease, particularly those related to dietary intake and malnutrition. Additionally, previous prospective studies have observed an independent association between malnutrition and decreased PCS and MCS only at baseline^(12,14)^ but did not assess the impact of malnutrition on change in HRQoL over time. We have now helped to fill this knowledge gap by showing that prevalence/development of malnutrition over 1 year was an independent predictor of the 1-year decrease in EQ5D HSS, and the 1-year decrease in fat intake (a measure of nutritional intake that contributes significantly to energy intake) was independently associated with the 1-year decline of MCS, PCS and EQ5D VAS. Inadequate dietary intake is an important marker of malnutrition and is associated with poor outcomes^(8)^. We observed that energy and protein intake were low at baseline compared with the recommended intake for persons receiving dialysis^(35)^. Lower protein intake correlated with lower PCS and EQ5D HSS at baseline and a decrease in energy intake over 1 year also correlated with the 1-year decrease in MCS and both EQ5D scores in univariable analyses but change in energy and protein intake did not enter the final multivariable models. These observations reinforce the need to conduct comprehensive nutritional screening and monitoring to identify those persons on dialysis at nutritional risk or already malnourished and then implement appropriate nutritional interventions to prevent malnutrition or improve nutritional status. This approach would be expected to improve HRQoL and clinical outcomes, though prospective clinical trials are warranted to test this hypothesis.

Similar to other studies conducted in dialysis populations^(2,16,24,36,37)^, we observed that diabetes, being on HD and unemployment status were independently associated with lower HRQoL scores at baseline. Also as reported in previous studies^(38,39)^, older age was an independent determinant of better MCS, PCS and EQ5D HSS. One possible explanation may be that older people are more accepting of the limitations caused by illness and have lower expectations of HRQoL, but this requires further investigation. Similar to our findings, previous studies have confirmed that low educational level and presence of CVD are independently associated with lower HRQoL scores^(40,41)^.

Several limitations need to be considered when interpreting our results. Owing to the observational nature of this study, we cannot infer a causal relationship between malnutrition and HRQoL. Prospective clinical trials will be needed to investigate this further. The relatively small sample size prevented us from including more potential determinants of HRQoL in multivariable analyses. This may in part account for the relatively low adjusted *R* ^2^ values in the multivariable analyses, suggesting the presence of residual confounding, and may have also resulted in a failure to detect associations between some variables and decrease in HRQoL scores. As this was a single centre study, our results cannot necessarily be extrapolated to other dialysis populations. Thus, larger multicentre studies are needed to confirm these findings. We did not use the Kidney Disease Quality of Life survey and therefore could not assess the impact of malnutrition on the kidney-specific QoL domains. However, the SF-36 questionnaire, which is included in the Kidney Disease Quality of Life survey as a generic chronic disease core component, is a widely used HRQoL instrument that has been validated in multicultural environments with large general population samples, as well as in persons receiving dialysis^(30)^. We acknowledge the use of multiple comparisons in our statistical analyses, and thus the borderline ‘significant’ associations that we observed could be due to chance. We have not adjusted *P*-values (e.g. Bonferroni correction)^(42–45)^ but have interpreted our results with caution in the light of multiple testing.

In conclusion, these findings strengthen the importance of undertaking nutritional screening and monitoring in all persons on dialysis to identify malnutrition, and providing specialised, individualised nutritional advice in order to prevent malnutrition and/or improve nutritional status. Further prospective clinical trials with larger sample sizes and longer follow-up are needed to evaluate the impact of dietetic interventions on HRQoL and other clinical outcomes.

## Supplementary material

For supplementary material accompanying this paper visit https://doi.org/10.1017/S000711452100249X. click here to view supplementary material
