# Supplementary material for: Impact of malnutrition on health-related quality of life in persons receiving dialysis: a prospective study
Source: Br J Nutr. 2021 Jul 5;127(11):1647–55. doi: 10.1017/S000711452100249X (PMC9201831; doi:10.1017/S000711452100249X)
Supplement: Supplementary file 1 [file S000711452100249Xsup001.docx]

*Supplementary Table 1.* Multivariable linear regression analysis to identify independent determinants of health-related quality of life at baseline (Model 2).

| *Independent variables* | *Dependent variable* | | | | | | | | | | | | | | | | | | | | | |
| --- | --- | --- | --- | --- | --- | --- | --- | --- | --- | --- | --- | --- | --- | --- | --- | --- | --- | --- | --- | --- | --- | --- |
|  | Mental component score | | | | | | Physical component score | | | | | EQ5D Health state score | | | | | | EQ5D Visual analogue score | | | | |
|  | B | | Beta | | p Value | | B | | Beta | | p Value | B | | | Beta | | p Value | B | | Beta | | p Value |
| Age (years) | 0.323 | 0.361 | | <0.0001 | | 0.054 | | 0.055 | | 0.5 | | | 0.004 | 0.215 | | 0.008 | | | 0.200 | 0.120 | 0.2 | |
| Sex (Male vs. Female) | 0.118 | 0.005 | | 1.0 | | 0.352 | | 0.013 | | 0.9 | | | -0.054 | -0.091 | | 0.3 | | | -3.226 | -0.068 | 0.5 | |
| Unemployed/retired (Yes vs. No) | -2.342 | -0.085 | | 0.3 | | -4.198 | | -0.140 | | 0.07 | | | -0.040 | -0.062 | | 0.4 | | | -9.390 | -0.182 | 0.04 | |
| Dialysis modality (PD vs. HD) | -0.374 | -0.013 | | 0.9 | | -4.546 | | -0.142 | | 0.04 | | | -0.109 | -0.159 | | 0.02 | | | -5.499 | -0.100 | 0.2 | |
| SGA score | 1.965 | 0.264 | | 0.002 | | 3.330 | | 0.411 | | <0.0001 | | | 0.073 | 0.423 | | <0.0001 | | | 4.866 | 0.351 | <0.0001 | |
| Diabetes (Yes vs. No) | -1.251 | -0.051 | | 0.5 | | -6.612 | | -0.247 | | 0.001 | | | -0.162 | -0.284 | | <0.0001 | | | -9.226 | -0.202 | 0.02 | |
| Dialysis vintage (months) | 0.013 | 0.071 | | 0.4 | | -0.017 | | -0.084 | | 0.2 | | | 0.000 | -0.061 | | 0.4 | | | 0.019 | 0.056 | 0.5 | |
| Handgrip strength (kg) | -0.013 | -0.013 | | 0.9 | | 0.140 | | 0.122 | | 0.2 | | | 0.004 | 0.151 | | 0.1 | | | -0.107 | -0.054 | 0.6 | |
| Adjusted R^2^ | 0.183 | | | | | 0.353 | | | | | | | 0.377 | | | | | | 0.179 | | | |

Results presented as unstandardized (B) and standardized (Beta) coefficients.

Abbreviations: EQ5D, European Quality of Life 5-Dimensions; HD, haemodialysis; PD, peritoneal dialysis; SGA, Subjective Global Assessment

*Supplementary Table 2.* Predictors of change in health-related quality of life scores over one year in univariable analysis.

| *Variable* | *Change in Mental Component Score* | | *Change in Physical Component Score* | | *Change in EQ5D Health State Score* | | *Change in EQ5D Visual Analogue Score* | |
| --- | --- | --- | --- | --- | --- | --- | --- | --- |
|  | *Increase/stable (n=74)* | *Decrease (n=43)* | *Increase/stable (n=75)* | *Decrease (n=42)* | *Increase/stable (n=59)* | *Decrease (n=58)* | *Increase/stable (n=65)* | *Decrease (n=52)* |
| *Sex*  Female (*n=*41)  Male (*n=*76) | 27 (66)  47 (62) | 14 (34)  29 (38) | 25 (61)  50 (66) | 16 (39)  26 (34) | 22 (54)  37 (49) | 19 (46)  39 (51) | 22 (54)  43 (57) | 19 (46)  33 (43) |
| *Diabetes*  Yes (*n=*51)  No (*n=*66) | 29 (57)  45 (68) | 22 (43)  21 (32) | 37 (73)  38 (58) | 14 (27)  28 (42) | 27 (53)  32 (48) | 24 (47)  34 (52) | 28 (55)  37 (56) | 23 (45)  29 (44) |
| *Coronary heart disease*  Yes (*n=*43)  No (*n=*74) | 22 (51)  52 (70) | 21 (49)*  22 (30) | 27 (63)  48 (65) | 16 (37)  26 (35) | 18 (42)  41 (65) | 25 (58)  33 (35) | 18 (42)  47 (63) | 25 (58)*  27 (37) |
| *Employed*  Yes (*n=*29)  No (*n=*88) | 20 (69)  54 (61) | 9 (31)  34 (39) | 21 (72)  54 (61) | 8 (28)  34 (39) | 17 (59)  42 (48) | 12 (41)  46 (52) | 14 (48)  51 (58) | 15 (52)  37 (42) |
| *Educational qualifications*  Yes (*n=*69)  No (*n=*48) | 44 (64)  30 (63) | 25 (36)  18 (37) | 46 (67)  29 (60) | 23 (33)  19 (40) | 40 (58)  19 (40) | 29 (42)  29 (60) | 45 (65)  20 (42) | 24 (35)*  28 (58) |
| *Dialysis modality*  Haemodialysis (*n=*93)  Peritoneal dialysis (*n=*24) | 60 (65)  14 (58) | 33 (35)  10 (42) | 58 (62)  17 (71) | 35 (38)  7 (29) | 48 (52)  11 (46) | 45 (48)  13 (54) | 54 (58)  11 (46) | 39 (42)  13 (54) |
| *1-year change energy intake*  Increase/stable (*n=*66)  Decrease (*n=*51) | 47 (71)  27 (53) | 19 (29)*  24 (47) | 46 (70)  29 (57) | 20 (30)  22 (43) | 39 (59)  20 (39) | 27 (41)*  31 (61) | 43 (65)  22 (43) | 23 (35)*  29 (57) |
| *1-year change protein intake*  Increase/stable (*n=*54)  Decrease (*n=*63) | 39 (72)  35 (56) | 15 (28)  28 (44) | 38 (70)  37 (59) | 16 (30)  26 (41) | 32 (59)  27 (43) | 22 (41)  36 (57) | 35 (65)  30 (48) | 19 (35)  33 (52) |
| *1-year change fat intake*  Increase/stable (*n=*64)  Decrease (*n=*53) | 47 (73)  27 (51) | 17 (27)*  26 (49) | 47 (73)  28 (53) | 17 (27)*  25 (47) | 37 (58)  22 (42) | 27 (42)  31 (58) | 43 (67)  22 (42) | 21 (33)*  31 (58) |
| Age (years) | 64 (IQR 55 to 75) | 66 (53 to 74) | 63 (54 to 73) | 68 (55 to 76) | 63 (53 to 73) | 67 (55 to 76) | 63 (53 to 75) | 67 (55 to 74) |
| 1-year Δ Haemoglobin (g/L) | -4.5 (-14.0 to 5.0) | -4.0 (-12.0 to 6.0) | -3.0 (-11.0 to 7.0) | -9.0 (-18.0 to 0.3)* | -3.0 (-14.0 to 5.0) | -4.0 (-12.3 to 4.0) | -5.0 (-14.0 to 6.0) | -3.5 (-12.0 to 4.0) |
| 1-year Δ C reactive protein (mg/L) | 0.3 (-5.0 to 3.0) | 0.0 (-3.1 to 7.3) | -0.1 (-4.8 to 2.5) | 1.0 (-3.0 to 8.0) | 0.0 (-3.0 to 4.0) | 0.0 (-5.0 to 7.0) | 0.0 (-6.0 to 8.0) | 0.0 (-3.1 to 2.4) |
| 1-year Δ Serum creatinine (µmol /L) | 4.5 (-64.5 to 117.5) | 27.0 (-87.0 to 73.0) | 23.0 (-36.0 to 96.0) | -15.5 (-118.3 to 120.0) | 25.0 (-62.0 to 132.0) | 3.0 (-86.3 to 81.8) | 25.0 (-76.5 to 122.5) | 4.5 (-61.5 to 72.3) |
| 1-year Δ Serum albumin (g/L) | 0.0 (-2.0 to 2.0) | -1.0 (-4.0 to 1.0) | 0.0 (-3.0 to 1.0) | -1.0 (-3.3 to 1.0) | 0.0 (-3.0 to 1.0) | -1.0 (-3.0 to 1.0) | 0.0 (-3.0 to 2.0) | -1.0 (-3.0 to 0.8) |
| 1-year Δ Serum total protein (g/L) | 0.5 (-3.0 to 4.0) | -1.0 (-5.0 to 1.0) | 1.0 (-4.0 to 3.0) | -1.0 (-4.3 to 1.0)* | 1.0 (-3.0 to 4.0) | -1.0 (-4.0 to 3.0) | 0.0 (-3.5 to 4.0) | -0.5 (-4.0 to 2.0) |
| 1-year Δ Body mass index (kg/m^2^) | -0.1 (-0.9 to 0.6) | -0.5 (-1.3 to 0.3) | -0.2 (-1.1 to 0.5) | -0.1 (-1.2 to 0.7) | -0.1 (-1.1 to 0.5) | -0.2 (-1.1 to 0.7) | -0.1 (-1.1 to 0.55) | -0.4 (-1.2 to 0.7) |
| 1-year Δ Handgrip strength (kg) | 0.2 (-1.9 to 2.9) | -1.8 (-3.9 to 2.1) | -0.5 (-2.3 to 2.9) | 0.4 (-3.2 to 2.2) | -0.1 (-3.1 to 3.2) | -0.5 (-2.3 to 2.3) | -0.6 (-2.9 to 3.1) | 0.4 (-2.3 to 2.2) |

Abbreviations: EQ5D, European Quality of Life 5-Dimensions; IQR, interquartile range.

Continuous variables expressed as median (interquartile range) and categorical variables expressed as numbers (percentage).

*p<0.05 Increase/stable vs. decrease in health-related quality of life scores.
